# Predictive Value of Elevated Neutrophil Gelatinase-Associated Lipocalin (NGAL) Levels for Assessment of Cardio–Renal Interactions among ST-Segment Elevation Myocardial Infarction Patients

**DOI:** 10.3390/jcm11082162

**Published:** 2022-04-13

**Authors:** David Zahler, Ilan Merdler, Ariel Banai, Eden Shusterman, Omri Feder, Tamar Itach, Leemor Robb, Shmuel Banai, Yacov Shacham

**Affiliations:** 1 Department of Cardiology, Tel-Aviv Sourasky Medical Center Affiliated to the Sackler Faculty of Medicine, Tel-Aviv University, Tel-Aviv 64239, Israel; david.zahler@gmail.com (D.Z.); ilanmerdler@gmail.com (I.M.); arielbanai@gmail.com (A.B.); itachtamar@gmail.com (T.I.); leemorbar@gmail.com (L.R.); shmuelb@tlvmc.gov.il (S.B.); 2 Internal Medicine Department H, Tel-Aviv Sourasky Medical Center Affiliated to the Sackler Faculty of Medicine, Tel-Aviv University, Tel-Aviv 64239, Israel; eden84@gmail.com (E.S.); omri.feder@gmail.com (O.F.)

**Keywords:** neutrophil gelatinase-associated lipocalin, ST-segment elevation myocardial infarction, acute kidney injury

## Abstract

Background: Elevated serum neutrophil gelatinase-associated lipocalin (NGAL) levels reflect both inflammatory reactions and renal tubular injury. Recently, associations with endothelial dysfunction and plaque instability were also proposed. We investigated the prognostic utility of elevated NGAL levels for renal and clinical outcomes among ST-segment elevation myocardial infarction (STEMI) patients treated with primary coronary intervention (PCI). Methods: We performed a prospective, observational, open-label trial. High NGAL was defined as values within the third tertile (>66 percentile). Results: A total of 267 patients were included (mean age 66 ± 14 years, 81% males). Short-term adverse outcomes were consistently increased in the high NGAL group with more acute kidney injury, lower mean left ventricular ejection fraction, higher 30-day mortality, and higher incidence for the composite outcome of major adverse cardiac events (MACE). In a multivariate logistic regression model, high NGAL emerged as a strong and independent predictor for MACE (OR 2.07, 95% CI 1.15–3.73, *p* = 0.014). Conclusions: Among STEMI patients undergoing primary PCI, elevated NGAL levels are associated with adverse renal and cardiovascular outcomes, independent of traditional inflammatory markers. Further studies are needed to assess the potentially unique role of NGAL in cardio–renal interactions.

## 1. Introduction

Cardio–renal interactions in acute myocardial infarction are complex and predict worse outcomes [1,2]. Neutrophil gelatinase-associated lipocalin (NGAL), a protein stored in both neutrophil blood cells and renal tubular cells, functions as an inflammatory modulator [3,4] and is an early and sensitive marker for acute kidney injury (AKI) [5,6,7].

Inflammatory biomarkers are known to rise and have significant predictive value for adverse outcomes in patients with acute coronary ischemia [8,9]. Indeed, higher plasma NGAL levels have been demonstrated among patients with acute myocardial infarction [10]. However, to date, only very few studies have assessed the prognostic value of NGAL in this setting [11,12]. We, therefore, examined the utility of NGAL and its independence from traditional inflammatory markers for the prognosis of adverse outcomes among ST-segment elevation myocardial infarction (STEMI) patients undergoing primary percutaneous coronary intervention (PCI).

## 2. Materials and Methods

### 2.1. Patients

A prospective, observational, open-label trial was performed in the Tel-Aviv Sourasky Medical Center. Included were STEMI patients admitted to the cardiac intensive care unit (CICU) following successful primary PCI between December 2017 and August 2021. As plasma NGAL in these patients can reflect chronic inflammation, we excluded patients with chronic inflammatory syndromes and malignancies (*n* = 19). No patients were lost to follow-up. The final study population included 267 STEMI patients. The diagnosis of STEMI was established by a typical history of chest pain, diagnostic electrocardiographic changes, and serial elevation of serum cardiac biomarkers [13]. Primary PCI was performed in patients with symptoms ≤12 h in duration as well as in patients with symptoms lasting 12–24 h in duration if the symptoms continued to persist at the time of admission. The contrast medium used in procedures was iodixanol (Visipaque, GE healthcare, Ireland). Following coronary interventional procedures, physiologic (0.9%) saline was given intravenously at a rate of 1 mL/kg/h for 12 h after contrast exposure. In patients with overt heart failure, the hydration rate was reduced at the discretion of the attending physician. All patients underwent a screening echocardiographic examination within 2 days of admission to assess left ventricular ejection fraction (LVEF). Patient records were further evaluated for the occurrence of adverse in-hospital outcomes. These included the development of heart failure (defined as the occurrence of both clinical and radiological signs of congestion), need for inotropic support, and 30-day mortality. Mortality up to 30 days following admission and after discharge was determined from computerized records of the population registry bureau. These three outcomes were united to form a composite outcome of major adverse cardiac events (MACE), as described previously [14]. The study was conducted according to the guidelines of the Declaration of Helsinki and approved by the Institutional Review Board (Name: Tel-Aviv Sourasky Medical Center Helsinki Committee, IRB code: TLV-16-0224). Informed consent was obtained from all subjects involved in the study.

### 2.2. Laboratory

Samples of venous blood for NGAL analysis were collected 24 h post-admission to the CICU for all patients. Samples were centrifuged within 10 min in a refrigerated centrifuge, and the plasma and serum were stored at −80 °C. NGAL was analyzed using NGAL rapid Elisa kits (Bioporto Diagnostics, Copenhagen, Denmark).

Serum creatinine (sCr) was determined upon hospital admission, prior to PCI, and at least once a day throughout hospitalization. AKI was determined using the KDIGO criteria and was defined as an increase in sCr ≥ 0.3 mg/dL within 48 h of admission or an increase in sCr ≥ 1.5 times baseline, which was known or presumed to have occurred within the prior 7 days [15]. Chronic kidney disease was categorized as admission estimated glomerular filtration rate (eGFR) of ≤60 mL/min/1.73 m^2^ [16].

Blood samples for C-reactive protein (CRP) levels and a complete blood count (CBC) were drawn following primary PCI. Quantitative CRP analysis was performed by the Bayer wide-range assay.

### 2.3. Statistics

Patients were stratified into two groups based on serum NGAL levels (ng/mL). Low NGAL levels were defined as values within the lower two tertiles (≤66 percentile), under an NGAL level of ≤109 ng/mL. High NGAL were defined as values within the highest tertile (>66 percentile), above an NGAL value of >109. Categorical variables were expressed as frequency and percentages. Normally distributed continuous variables were described using mean and standard deviation (SD) and non-normal ones with median (interquartile range 25–75%). Chi-square test was used to evaluate associations between categorical variables. Continuous variables were compared using the independent samples *t*-test or the Mann–Whitney U test, depending on the distribution. The predictive value of NGAL for the risk of MACE was evaluated with multivariate binary logistic regression models adjusted for other inflammatory laboratory variables. Adjusted odds ratios (OR) with a 95% confidence interval (95% CI) were reported for all variables. To further investigate the predictive power of NGAL, receiver operating characteristic (ROC) curves were constructed. Area under the curves (AUC) with corresponding 95%CI were reported for relevant inflammatory markers and compared using the Delong and Delong method [17]. A two-tailed *p*-value of <0.05 was considered significant for all analyses. Analyses were performed with the IBM SPSS software (SPSS Inc., Chicago, IL, USA).

## 3. Results

A total of 267 patients were included (mean age 66 ± 14 years, 81% males); of them, 89 (33%) were defined as the high NGAL group. Median total NGAL levels were 90 (65–124) ng/mL, 76 (59–90) ng/mL in the low NGAL group as compared to 141 (123–183) ng/mL for the high NGAL group (*p* < 0.0001).

Baseline characteristics are shown in Table 1. Patients with high NGAL values were older and more likely to be female, have hypertension, or have a prior myocardial infarction. They were less likely to have a family history of coronary artery disease. Patients in the high NGAL group had more chronic kidney disease and lower mean eGFR rates. As displayed in Table 2, the high NGAL group had higher admission CRP values (14.5 (13.4–15.5) mg/L vs. 7.3 (2.5–18.7) mg/L, *p* = 0.004) but similar white blood cell counts (WBC) (10.5 (8.4–13.2) 10^9^/L vs. 10.9 (8.5–12.9) 10^9^/L, *p* = 0.58). Admission hemoglobin and sCR levels differed between both groups as well.

**Figure 1:**
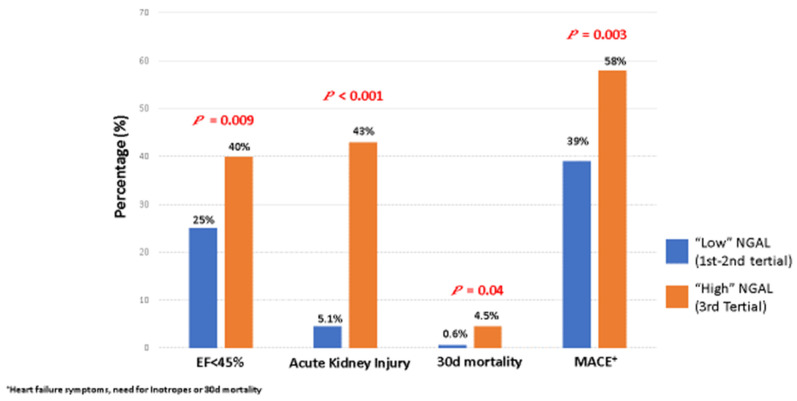
Adverse short-term clinical outcomes stratified by NGAL levels; ^+^ clinical heart failure findings, need for inotropes or 30-day mortality; NGAL, neutrophil gelatinase-associated lipocalin.

As presented in Table 3, adverse outcomes were consistently more common in the high NGAL group, including lower mean LVEF (46 ± 8% vs. 44 ± 9%, *p* = 0.04), higher incidence of periprocedural bleeding (6 (3.4%) vs. 9 (10.1%), *p* = 0.02), and a longer length of hospitalization (4 (3–5) days vs. 5 (4–7) days, *p* < 0.001). Incidence of AKI (9 (5.1%) vs. 38 (43%), *p* < 0.001), 30-day mortality (1 (0.6%) vs. 4 (4.5%), *p* = 0.04), and the composite outcome of MACE (70 (39)% vs. 52 (58)%, *p* = 0.003) were all significantly increased in the high NGAL group (Figure 1). In multivariate binary logistic regression models, NGAL emerged as a strong independent predictor for MACE (Table 4). This was observed for both NGAL as a continuous variable (OR for each 100 ng/mL increase 2.07, 95% CI 1.15–3.73, *p* = 0.014) and when defined as a high vs. low binary variable (OR 2.09, 95% CI 1.04–4.19, *p* = 0.038). Other independent predictors were baseline chronic kidney disease and occurrence of acute kidney injury during hospitalization.

**Figure 2:**
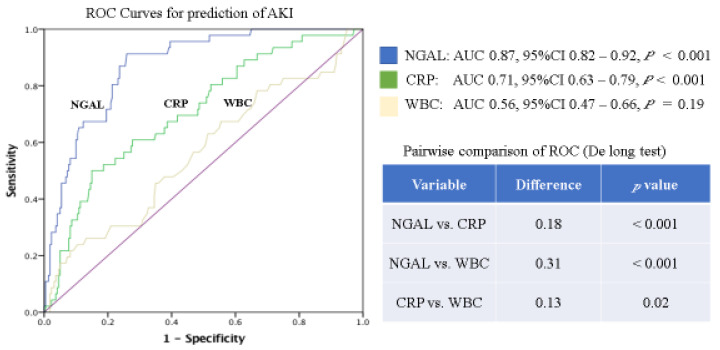
ROCs and corresponding AUCs of inflammatory laboratory markers for prediction of AKI; NGAL, neutrophil gelatinase-associated lipocalin; CRP, C-reactive protein; WBC, white blood cells.

**Figure 3:**
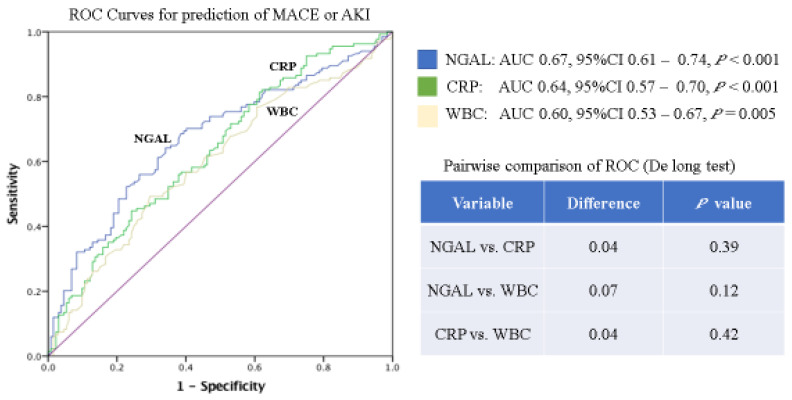
ROC and corresponding AUCs of inflammatory laboratory markers for prediction of the combined endpoint: AKI or MACE; NGAL, neutrophil gelatinase-associated lipocalin; CRP, C-reactive protein; WBC, white blood cells.

Furthermore, as demonstrated by the ROC in Figure 2, NGAL had higher AUCs (NGAL 0.87 vs. CRP 0.71 vs. WBC 0.56) with a significant advantage over the other inflammatory markers for the prediction of AKI (*p* < 0.001). The ROCs for the prediction of the combined outcome of AKI or MACE showed a higher AUC for NGAL (NGAL 0.67 vs. CRP 0.64 vs. WBC 0.60); however, these differences (NGAL vs. CRP *p* = 0.39, NGAL vs. WBC *p* = 0.12) did not reach statistical significance (Figure 3).

## 4. Discussion

The results of this study provide more evidence for the unique value of NGAL in the assessment of cardio–renal interactions. Our results demonstrate that NGAL has an independent value not only for renal outcomes but additionally may predict concomitant cardiovascular outcomes in acute myocardial infarction.

NGAL, a 25-kDa protein covalently bound to gelatinase proteins in human neutrophils, was reported as an early marker of kidney tubular injury in various patient populations and hence was nicknamed “kidney troponin.” It is released by renal tubular cells in cases of acute tubular damage and may be detected within a few hours of the tubular insult and even in the absence of functional AKI, manifested by serum creatinine elevation [7]. Previous studies have shown the value of serum NGAL for the early diagnosis of AKI in various patient populations, including critically ill, sepsis, post-surgery, and coronary angiography [18,19].

Cardiorenal interactions are complex and often bidirectional in nature. The typical insult in STEMI is Type 1 cardiorenal syndrome, in which rapid worsening of cardiac function leads to acute kidney injury [1]. NGAL is one of the earliest markers for this type of derangement [20,21]. Additionally, NGAL released by neutrophils also plays a role in rapid inflammatory modulations [3]. Both rapid inflammatory reactions and early renal dysfunction are important prognostic markers in acute coronary syndromes [2,8].

Besides the well-described associations with inflammation and kidney function, NGAL is also produced by cardiomyocytes and increased gene expressions were observed in various pathological cardiac processes such as chronic heart failure [18], myocarditis [22], and chronic coronary artery disease [23], with even higher levels in acute myocardial infarction probably reflecting an ischemic inflammatory reaction [10]. Furthermore, by modulating the function of matrix metalloproteinases [24], NGAL has been proposed to affect plaque instability [25], potentially affecting ischemic event severity or the risk for future coronary events. All these points underline the predictive potential of NGAL in acute coronary syndrome cascades.

However, the elevation of traditional markers of inflammation such as CRP and WBC levels are also associated with an increased risk of heart failure and mortality after acute myocardial infarction [8,26,27]. In addition, following STEMI, early sCR changes correlate with both short- and long-term mortality [28]. Nevertheless, the close, established, direct concomitant relationships to both renal and inflammatory processes may point to a unique prognostic utility for NGAL in acute coronary events.

In a study by Li et al., NGAL levels had a better ability to discriminate patients with severe coronary stenosis as compared to other inflammatory markers such as CRP [29]. In another study including individuals without known cardiovascular disease, NGAL predicted cardiovascular mortality independently of traditional risk factors such as renal function and biomarkers such as CRP [30].

However, other investigators have argued that these associations are largely mediated by leukocytosis, stating the lack of data regarding leukocyte counts in some publications [31,32]. Other studies have proposed that NGAL’s prognostic significance on mortality in coronary artery disease is mainly derived from concomitant higher neutrophil counts and that it is not independent of renal function [33], concluding that NGAL may not have any additional independent value for myocardial infarction patients.

Only a few studies have assessed the value of NGAL in STEMI patients undergoing PCI. A small-scale study showed higher MACE and mortality with increasing NGAL levels [11]. Helanova et al. demonstrated that the addition of NGAL to the TIMI score significantly improved risk stratification [34]. However, both studies did not assess its independent value to other inflammatory markers. Furthermore, our study demonstrated that NGAL levels have a prognostic value in STEMI patients, regardless of the development or exacerbation of renal failure, as shown in a regression model adjusted for both chronic renal failure and AKI.

A study published by Lindberg et al. [12] demonstrated that in STEMI patients, high NGAL levels were associated with all-cause mortality and MACE compared with patients with low NGAL levels. In multivariate models, the effect was independent of renal function and CRP levels. WBC counts were not included in the models.

Our results are consistent with the study by Lindberg et al. [12]. However, as opposed to previous arguments [31], we further focused on demonstrating independence from both humoral and cellular immune components. This effect may well be explained by the various additional effects of NGAL on endothelial and renal functions.

Our study bears notable limitations. This was a single-center study, and the number of patients recruited represents a modest sample size. We used sCr and eGFR as surrogate markers of kidney function, knowing that these markers have limitations when used in acute hospitalized patients with STEMI. In addition, no information was present on contrast media volume. Further investigations are needed before firm conclusions about the clinical implications of NGAL measurements can be drawn.

## 5. Conclusions

Among STEMI patients undergoing primary PCI, NGAL levels are associated with adverse renal and clinical outcomes, independent of traditional inflammatory markers such as WBC counts and CRP. Further investigations assessing the possible role of NGAL in cardio–renal interactions are warranted.

## Figures and Tables

**Table 1:** Baseline characteristics of 267 STEMI patients stratified by NGAL levels.

	Low NGAL (1st–2nd Tertile) (*n* = 178)	High NGAL (3rd Tertile) (*n* = 89)	*p*-Value
Age (years), mean ± SD	63 ± 13	74 ± 13	<0.001
Gender (female), *n* (%)	27 (15)	25 (28)	0.012
Hypertension, *n* (%)	92 (52)	69 (78)	<0.001
Diabetes mellitus, *n* (%)	60 (34)	31 (35)	0.86
Hyperlipidemia, *n* (%)	106 (60)	59 (66)	0.28
Family history of CAD, *n* (%)	39 (22)	6 (7)	0.002
Smoking history, *n* (%)	82 (46)	36 (40)	0.38
Multivessel coronary artery disease (>1 vessel), *n* (%)	112 (63)	51 (57)	0.37
Past myocardial infarction, *n* (%)	39 (22)	38 (43)	<0.001
Chronic kidney disease, *n* (%)	33 (19)	57 (64)	<0.001
eGFR (ml/minute/1.73^2^), mean ± SD	86 ± 25	56 ± 25	<0.001

NGAL, neutrophil gelatinase-associated lipocalin; CAD, coronary artery disease; eGFR, estimated glomerular filtration rate.

**Table 2:** Laboratory parameters stratified by NGAL levels.

	Low NGAL (1st–2nd Tertile) (*n* = 178)	High NGAL (3rd Tertile) (*n* = 89)	*p*-Value
Peak troponin (ng/L), median (IQR)	23,449 (10,297–70,070)	16,061 (1754–90,629)	0.18
Admission CRP (mg/L), median (IQR)	4.4 (1.7–10.0)	7.3 (2.5–18.7)	0.004
Admission hemoglobin (g/dL), median (IQR)	14.5 (13.4–15.5)	13.5 (11.9–14.7)	<0.001
Admission WBC (10^9^/L), median (IQR)	10.5 (8.4–13.2)	10.9 (8.5–12.9)	0.58
Admission creatinine (mg/dL), median (IQR)	0.92 (0.81–1.04)	1.3 (1.0–1.65)	<0.001

NGAL, neutrophil gelatinase-associated lipocalin; CRP, C-reactive protein; WBC, white blood cells. IQR, interquartile range.

**Table 3:** Adverse short-term outcomes stratified by NGAL levels.

	Low NGAL (1st–2nd Tertile) (*n* = 178)	High NGAL (3rd Tertile) (*n* = 89)	*p*-Value
AKI, *n* (%)	9 (5.1)	38 (43)	<0.001
LVEF (%), mean ± SD	46 ± 8	44 ± 9	0.04
LVEF <45%, *n* (%)	44 (25)	36 (40)	0.009
Length of stay (days), median (IQR)	4 (3–5)	5 (4–7)	<0.001
Cardiogenic shock/pulmonary edema, *n* (%)	6 (3.4)	6 (6.7)	0.22
Periprocedural bleeding, *n* (%)	6 (3.4)	9 (10.1)	0.02
30-day mortality, *n* (%)	1 (0.6)	4 (4.5)	0.04
MACE ^+^, *n* (%)	70 (39)	52 (58)	0.003
MACE ^+^ or LVEF <45%, *n* (%)	81 (46)	55 (62)	0.01

^+^ Clinical heart failure findings, need for inotropes or 30-day mortality; NGAL, neutrophil gelatinase-associated lipocalin; AKI, acute kidney injury; LVEF, left ventricular ejection fraction; MACE, major adverse cardiac events. IQR, interquartile range.

**Table 4:** Multivariate binary logistic regression analysis for prediction of MACE ^+^.

	Model 1		Model 2	
	OR (95% CI)	*p*-Value	OR (95% CI)	*p*-Value
NGAL (100 ng/mL)	2.07 (1.15–3.73)	0.014		
High NGAL (3rd Tertial)			2.09 (1.04–4.19)	0.038
Age (years)	1.01 (0.97–1.03)	0.76	1.01 (0.97–1.03)	0.89
Gender (male)	0.98 (0.47–2.07)	0.97	1.01 (0.48–2.12)	0.98
Diabetes mellitus	0.64 (0.35–1.74)	0.15	0.71 (0.38–1.29)	0.26
Hypertension	1.28 (0.67–2.44)	0.45	1.29 (0.67–2.45)	0.45
Hyperlipidemia	0.85 (0.47–1.54)	0.59	0.88 (0.49–1.59)	0.67
Family history of CAD	0.79 (0.36–1.74)	0.56	0.76 (0.35–1.67)	0.49
Smoking history	0.78 (0.44–1.39)	0.41	0.79 (0.45–1.39)	0.42
Past myocardial infarction	0.68 (0.35–1.31)	0.25	0.68 (0.35–1.31)	0.24
Chronic kidney disease (eGFR < 60)	0.36 (0.15–0.85)	0.02	0.38 (0.16–0.89)	0.03
Acute kidney injury	3.35 (1.35–8.28)	0.009	3.67 (1.51–8.98)	0.004
Baseline CRP (mg/L)	1.01 (0.99–1.02)	0.28	1.01 (0.99–1.02)	0.35
White blood cells (10^9^/L)	1.08 (0.99–1.15)	0.054	1.09 (1.01–1.17)	0.03

^+^ Clinical heart failure findings, need for inotropes or 30-day mortality; NGAL, neutrophil gelatinase-associated lipocalin; CAD, coronary artery disease; eGFR, estimated glomerular filtration rate; CRP, C-reactive protein.

## Data Availability

The data presented in this study are available on request from the corresponding author.
